# The biological basis and function of *GNAS* mutation in pseudomyxoma peritonei: a review

**DOI:** 10.1007/s00432-020-03321-8

**Published:** 2020-07-22

**Authors:** Yu-Lin Lin, Ru Ma, Yan Li

**Affiliations:** Department of Peritoneal Cancer Surgery and Pathology, Beijing Shijitan Hospital, Capital Medical University, No. 10 Tieyi Road, Yangfangdian Street, Haidian District, Beijing, 100038 China

**Keywords:** Pseudomyxoma peritonei, *GNAS*, Gene mutation, Signaling pathway, Mucin

## Abstract

**Purpose:**

Pseudomyxoma peritonei (PMP) is a rare clinical malignancy syndrome characterized by the uncontrollable accumulation of copious mucinous ascites in the peritoneal cavity, resulting in “jelly belly”. The mechanism of tumor progression and mucin hypersecretion remains largely unknown, but *GNAS* mutation is a promising contributor. This review is to systemically summarize the biological background and variant features of *GNAS*, as well as the impacts of *GNAS* mutations on mucin expression, tumor cell proliferation, clinical-pathological characteristics, and prognosis of PMP.

**Methods:**

NCBI PubMed database (in English) and WAN FANG DATA (in Chinese) were used for literature search. And NCBI Gene and Protein databases, Ensembl Genome Browser, COSMIC, UniProt, and RCSB PDB database were used for gene and protein review.

**Results:**

*GNAS* encodes guanine nucleotide-binding protein α subunit (Gsα). The mutation sites of *GNAS* mutation in PMP are relatively stable, usually at Chr20: 57,484,420 (base pair: C-G) and Chr20: 57,484,421 (base pair: G-C). Typical *GNAS* mutation results in the reduction of GTP enzyme activity in Gsα, causing failure to hydrolyze GTP and release phosphoric acid, and eventually the continuous binding of GTP to Gsα. The activated Gsα could thus continuously promote mucin secretion through stimulating the cAMP-PKA signaling pathway, which is a possible mechanism leading to elevated mucin secretion in PMP.

**Conclusion:**

*GNAS* mutation is one of the most important molecular biological features in PMP, with major functions to promote mucin hypersecretion.

## Introduction

Pseudomyxoma peritonei (PMP) is a rare clinical malignancy syndrome usually caused by the perforation of appendiceal mucinous tumor and the “redistribution phenomenon” of mucus and tumor cells, with an incidence of 1–2/million (Mittal et al. 2017; Smeenk et al. 2008). PMP is characterized by a large volume of mucinous ascites, multiple peritoneal implantations, omental cake, and ovarian involvement in women macroscopically, and abundant mucus pools microscopically. The chronic and uncontrollable mucus accumulation is one of the major clinical features of PMP (O’Connell et al. 2002a, b), which gradually leads to intraperitoneal organ adhesion, bowel obstruction, malnutrition, and eventually cachexia and death. Aggressive cytoreductive surgery (CRS) combined with hyperthermic intraperitoneal chemotherapy (HIPEC) could bring significant survival benefit to PMP (Chua et al. 2012; Li et al. 2018), and has been recommended by Peritoneal Surface Oncology Group International (PSOGI) as the standard treatment of PMP (Li et al. 2014, 2019).

Although treated with CRS plus HIPEC, patients frequently suffered from relapse, presenting aggravated “jelly belly”. One of the difficulties in studying PMP is the scarcity of knowledge in the fundamental molecular mechanisms underlying mucus hypersecretion. It has been reported that Kirsten rat sarcoma viral oncogene homolog (*KRAS*) and guanine nucleotide-binding protein alpha subunit (*GNAS*) are two of the most frequently detected variants in PMP, and *GNAS* mutation plays an important role in the regulation of mucin expression (Bradbury 2000; Jarry et al. 1994; Nishikawa et al. 2013). To have a better insight into the role of *GNAS* gene in PMP, we systemically reviewed the biological background of *GNAS*, current studies concerning the variant feature of *GNAS*, the impacts of *GNAS* mutations on mucin expression, tumor cell proliferation, and clinical–pathological characteristics and prognosis.

## The biological background of *GNAS* gene

### Basic structure and function

**Fig. 1:**
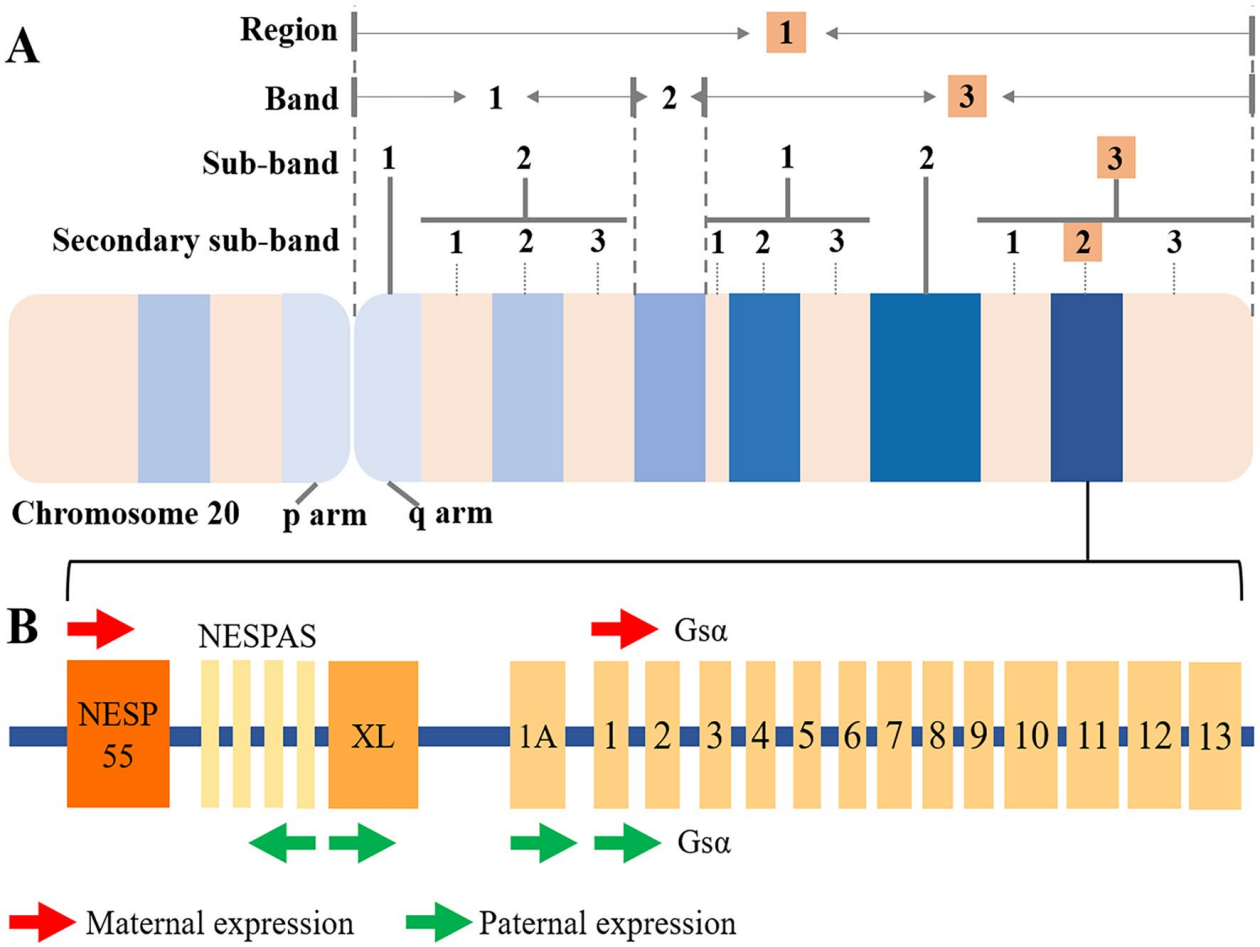
The location and biological structure of *GNAS* gene. **a***GNAS* gene is located at chromosome 20q13.32; **b** thirteen exons and the upstream alternative first exons of *GNAS*. The imprinted expression pattern of *GNAS* is highly complicated, with exclusively maternal expression of NESP55 (red arrow), and exclusively paternal expression of NESPAS, XL exon and exon 1A (green arrow). NESPAS: NESP anti sense

The *GNAS* gene is located at chromosome 20q13.32 (chromosome 20: 57,414,773–57,486,247), which also names *GNAS* complex locus (Fig. 1a), consisting of 13 exons and 12 introns. *GNAS* is responsible for the encoding of stimulatory guanine nucleotide-binding protein (G protein) α subunit (Gsα), which transduces signals from G protein-coupled receptors (GPCR) to adenyl cyclase (AC), and finally regulates the expression of cyclic adenosine monophosphate (cAMP).

### DNA transcription and translation

The promoter region of Gsα is located at the CpG island upstream of exon 1, which is usually unmethylated in alleles of both parental origins (Bird 1986; Gardiner-Garden and Frommer 1987). It was reported by Mantovani et al. (2002) and Germain-Lee et al. (2005) that Gsα imprinted with tissue-specific pattern in kidney cortex, thyroid gland, pituitary gland, and ovary, which is mainly maternally expressed. There are four kinds of alternative promoter regions upstream of Gsα exon 1 (Weinstein et al. 2001): (1) promoter 1, about 49 kb upstream of Gsα exon 1, encodes neuroendocrine secretory protein 55 (NESP55). The coding sequence is within the upstream of Gsα exon 1, leaving exon 2–13 untranslated region; (2) promoter 2, about 2–3 kb upstream of XL exon, initiates NESP55 exon transcription from the opposite direction; (3) promoter 3, about 35 kb upstream of Gsα exon 1, encodes extra-large alphas protein (XLαs), whose coding sequence is composed of XL exon and Gsα exon 1; (4) promoter 4 locates at about 2.5 kb upstream of Gsα exon 1. The resulted exon 1A transcripts were presumed to be untranslated mRNAs. The imprinted expression patterns of the aforementioned promoters are highly complicated. NESP55 is maternally expressed, while NESP anti sense, XLαs, and exon 1A are paternally expressed (Fig. 1b) (Crane et al. 2009).

The UniProt database (https://www.uniprot.org/) was used to search for proteins encoded by *GNAS*, with the searching term as “gene: GNAS AND reviewed: yes AND organism: “Homo sapiens (Human) [9606]””. The result showed four kinds of proteins encoded by *GNAS*: (1) Gsα, with a length of 394 amino acid residues, is encoded by *GNAS* exon 1–13; (2) XLαs, with a length of 1037 amino acid residues, is paternally expressed and responsible for the stimulation of AC-cAMP–PKA signaling pathway. XLαs is one of the isoforms of Gsα, with similar downstream receptor to Gsα. But there is no evidence showing that seven-transmembrane receptors activating Gsα can also activate XLαs; (3) protein ALEX, with a length of 626 amino acid residues, is the product of paternal expression of XL exon and possibly contributes to the inhibition of AC activity in XLαs subunit (Abramowitz et al. 2004); (4) NESP55, with a length of 245 amino acids, is maternally expressed and encoded by NESP55 exon. NESP55 forms LHAL tetrapeptide and GPIPIRRH peptide after modification and shear.

### The structure and function of Gsα

**Fig. 2:**
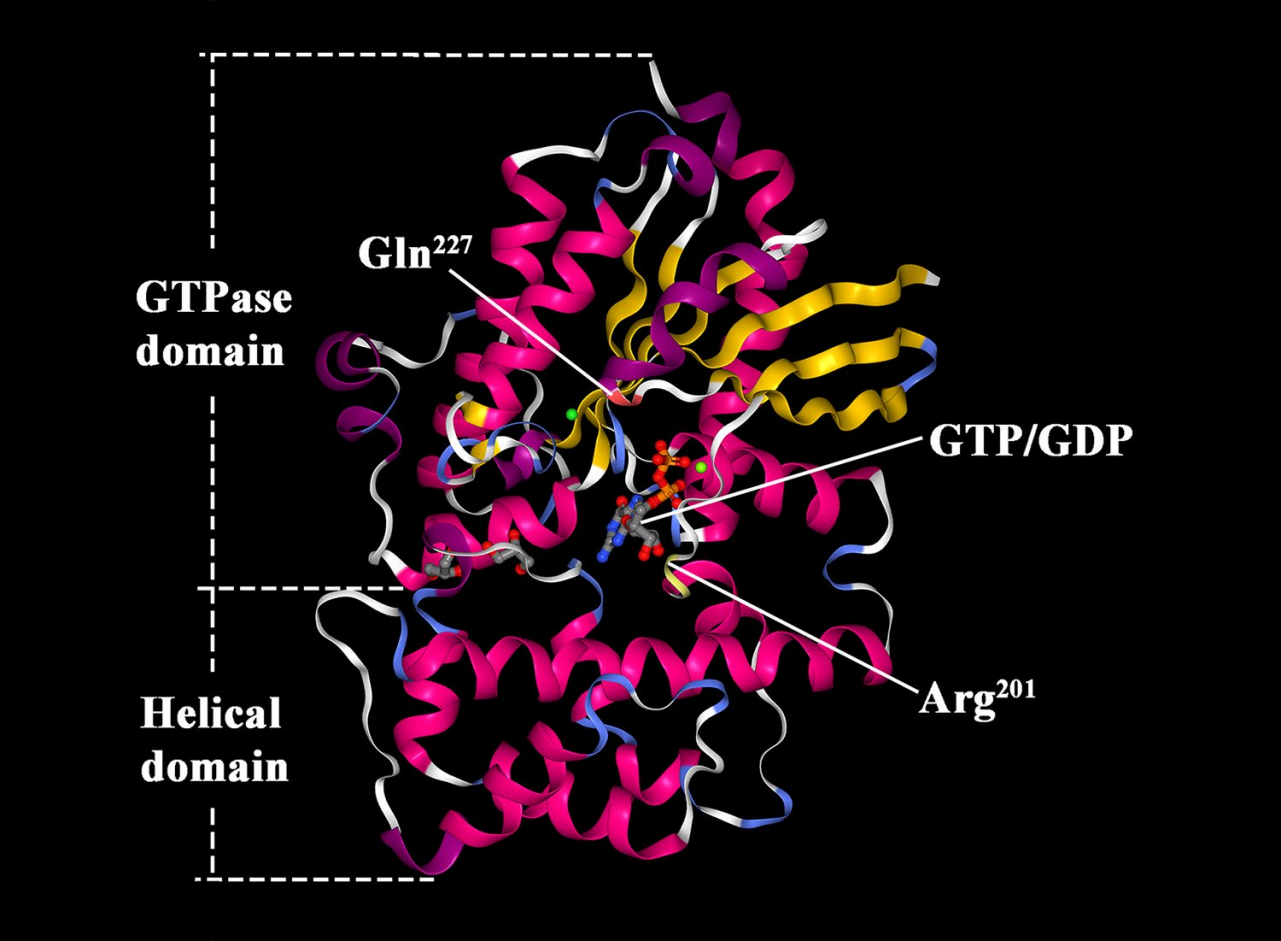
The tertiary structure of Gsα subunit (cited from RCSB PDB database, https://www.rcsb.org/; PDB ID: 6AU6). Magenta and purple: α-helix; yellow and blue: β-sheet; white: random coil. *Arg* arginine, *Gln* glutamine, *GTP* guanosine triphosphate, *GTPase* GTP enzyme, *GDP* guanosine diphosphate

Among the four reviewed proteins, Gsα is the main product of *GNAS* gene, which includes two domains (Rose et al. 2018) (Fig. 2). The first is guanosine triphosphate enzyme (GTPase) domain, which is formed after the fold of 39–394th amino acid residues. GTPase domain functions as the guanosine-biding and interaction site for receptors and effectors. There are four guanosine triphosphate/guanosine diphosphate (GTP/GDP)-binding sites, located at 47–55th, 197–204th, 223–227th, and 292–295th amino acid residues respectively; and two magnesium ion-binding sites, located at 54th and 204th amino acid residues, respectively. Two out of the four GTP/GDP-binding sites are highly conserved [arginine^201^ (Arg^201^) and glutamine^227^ (Gln^227^)], which play a vital role on the hydrolysis of the bound GTP. The second is helical domain, with a possible function of maintaining the binding status between GTP/GDP and Gsα (Weinstein et al. 2001). Besides the four domains, there are five motif structures in Gsα, including G1 (42–55th amino acids), G2 (196–204th amino acids), G3 (219–228th amino acids), G4 (288–295th amino acids), and G5 (364–369th amino acids).

**Fig. 3:**
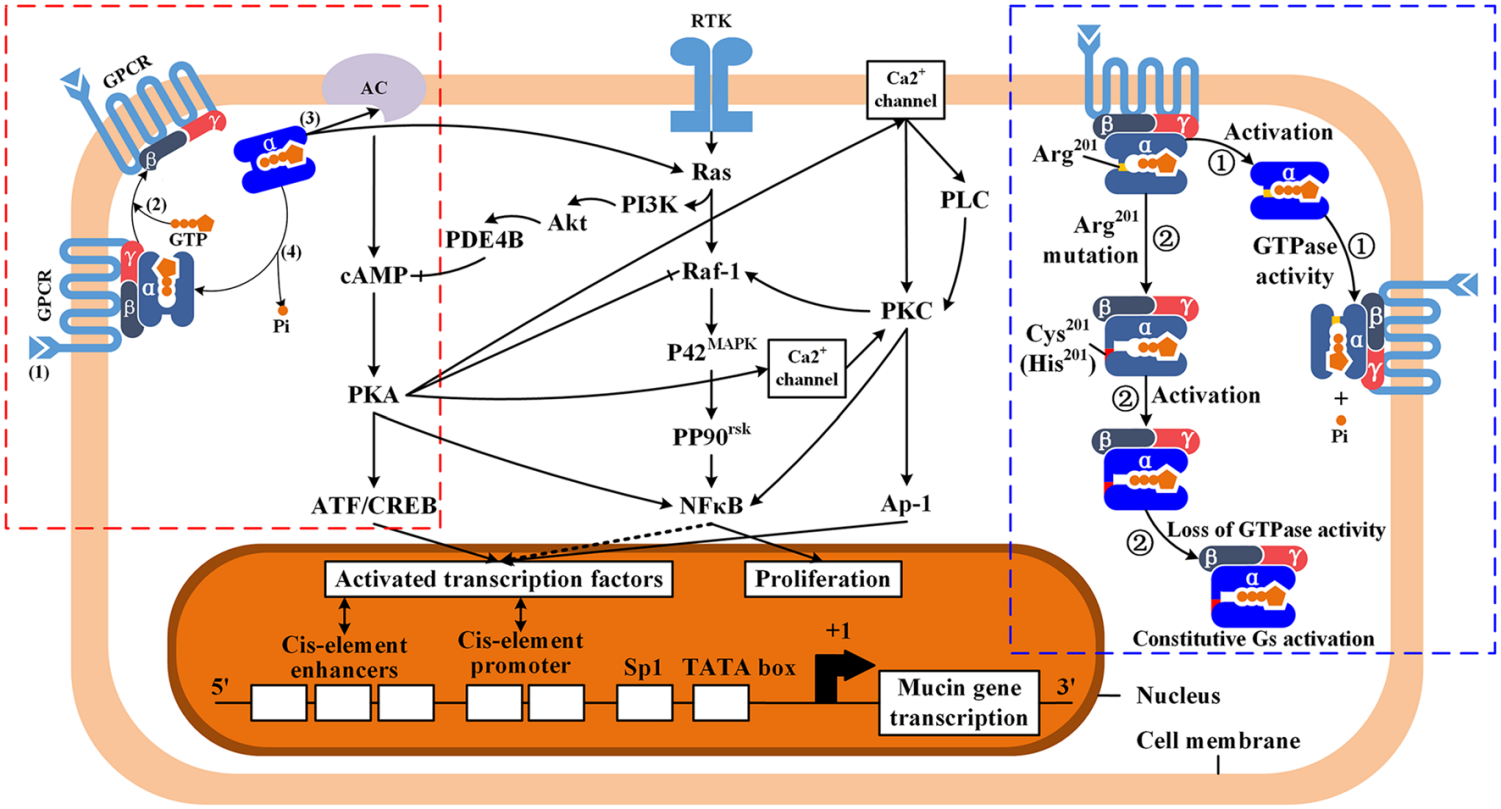
G protein cycle, activity changes of Gsα subunit caused by *GNAS* mutation, and the crosslink among Gsα subunit-induced cAMP–PKA, PI3K–Akt, and RAS–MAPK pathways. Red dotted box: G protein cycle; blue dotted box: activity changes of Gsα subunit caused by the mutation of Gsα Arg^201^. ① In the physiological status, activated Gsα returns to deactivated status after releasing a Pi; ② In the situation of Arg^201^ mutation, Gsα fails to release Pi and remains in activated status. Gsα in ink blue: deactivated status. Gsα in light blue: activated status; the other signaling pathways: cAMP–PKA, PI3K–Akt, and RAS–MAPK pathways interacts among each other and eventually modulate mucin gene expression via the nuclear import of ATF/CREB and NFκB. *GPCR* G protein-coupled receptor, *Gsα* stimulatory G protein subunit, *Pi* inorganic phosphate, *AC* adenyl cyclase, *cAMP* cyclic adenosine monophosphate, *PKA* protein kinase A, *ATF* activating transcription factor, *CREB* cAMP-response element-binding protein, *PLC* phospholipase C, *PKC* protein kinase C, *PI3K* phosphoinositide 3-kinase, *Akt* protein kinase B, *PDE4B* phosphodiesterase 4B, *RTK* receptor tyrosine kinases, *Ras* rat sarcoma protein, *Raf-1* Raf-1 protein, *P42*^*MAPK*^ P42 mitogen-activated protein kinas, also named Erk2, extracellular signal-regulated kinase 2, *PP90*^*rsk*^ 90 kDa ribosomal S6 kinase, *NFκB* nuclear factor kappa-light-chain-enhancer of activated B cells, *Sp1* specificity protein 1, *Arg* arginine, *Cys* cysteine, *His* histidine

The signaling from GPCR to the downstream molecules is carried out through G protein cycle (Fig. 3, red-dotted box): (1) Gsα releases GDP and combines with GTP due to the affinity reduction between Gsα and GDP caused by activation from ligand-binding GPCR to Gsα; (2) GTP-binding Gsα separates with β and γ subunits and turns into an activated status, which is able to stimulate downstream molecules; (3) as reacting with the downstream molecules, the GTPase activity of Gsα is activated and then GTP is hydrolyzed. Eventually, Gsα returns to the primary structure and reforms trimer with β and γ subunits.

## The molecular changes of *GNAS* mutation

A thorough literature research identified 13 papers reporting the genetic variants and corresponding gene mutation rates in PMP. Only variants reported in ≥ 5 papers were listed in Table 1. As listed in Table 1, the two most frequent variants in PMP are *KRAS* and *GNAS* mutations, with a median mutation rates of 77.8% (range 40.0–100%) and 45.7% (range 25.7–100%) respectively. By reviewing papers describing the detailed variant form of *GNAS*, we found that the most frequently detected *GNAS* mutation forms were c.602G>A (p.R201H) and c.601C>T (p.R201C) (Table 2). Despite the different variant forms reported by Pengelly et al. (2018) and Saarinen et al. (2017), the variant sites were relatively stable, both located at Chr20: 57,484,420 and Chr20: 57,484,421, which was identical to c.602G>A (p.R201H) and c.601C>T (p.R201C). Various transcripts chosen after sequencing might have resulted in the different expression patterns of mutation sites. Thus, it can be concluded that Chr20: 57,484,420 C>T (c.601C>T: p.R201C) and Chr20: 57,484,421 G>A (c.602G>A: p.R201H) are the two most significant variant forms in PMP *GNAS* mutations.

**Table 1:** Summary of the top 5 mutations in pseudomyxoma peritonei

References	Cases	Gene panel	Gene mutation rates (%)						
			*KRAS*	*GNAS*	*KRAS* + *GNAS*	*TP53*	*SMAD4*	*APC*	*PI3CA*
Tokunaga et al. (2019)	183	592	55.0	31.0	NA	40.0	16.0	10.0	6.0
Pengelly et al. (2018)	5	54	100.0	100.0	100.0	NA	10.0	10.0	NA
Gleeson et al. (2018)	19–31^a^	47	80.6	73.7	87.0	5.0	16.0	11.0	10.0
Saarinen et al. (2017)	9	Whole exome	100.0	55.6	55.6	NA	NA	NA	NA
Borazanci et al. (2017)	116–396^a,b^	47	57.3	28.2	NA	23.4	16.2	10.7	5.3
Pietrantonio et al. (2016a, b)	40	50	72.0	52.5	NA	12.5	2.5	NA	7.5
Nummela et al. (2015)	19	48	100.0	63.2	NA	5.3	15.3	0.0	5.3
Noguchi et al. (2015)	18	50	77.8	44.4	NA	22.2	16.7	NA	11.1
Sio et al. (2014)	10	236	70.0	40.0	40.0	NA	NA	NA	NA
Liu et al. (2014)	35	50	42.9	25.7	NA	20.0	14.3	22.9	5.7
Alakus et al. (2014)	29	NA	89.7	70.0	NA	0.0	NA	NA	0.0
Singhi et al. (2014)	55	2	40.0	31.0	NA	NA	NA	NA	NA
Nishikawa et al. (2013)	35	2	94.3	45.7	42.9	NA	NA	NA	NA
Range	NA	NA	40.0–100.0	25.7–100.0	40.0–100.0	0–40.0	2.5–16.7	0–22.9	0–11.1
Median	NA	NA	77.8	45.7	55.6	16.3	15.7	10.4	5.9

*NA* not available

^a^Number of patients varied by different genes detected

^b^Patients with neuroendocrine tumors of appendix were excluded

**Table 2:** The variant forms of *GNAS* mutation

References	Cases	Gene panel	*GNAS* mutation, *N*	*GNAS* mutation rate	*GNAS* variant form (%)							
					c.602G>A (p.R201H)	c.601C>T (p.R201C)	c.601C>A (p.R201S)	p.Q227STOP	p.Q227H	c.C556T (p.R186C)	c.G557A (p.R186H)	c.G560A (p.R187H)
Tokunaga et al. (2019)	183	592	57	31.0	NA	NA	NA	NA	NA	NA	NA	NA
Pengelly et al. (2018)	5	54	5	100.0	0	0	0	0	0	20.0 (1/5)	30.0 (3/5)	20.0 (1/5)
Gleeson et al. (2018)	19	47	14	73.7	54	46	0	0	0	0	0	0
Saarinen et al. (2017)	9	Whole exome	5	55.6	NA	NA	NA	NA	NA	NA	NA	NA
Borazanci et al. (2017)	124	47	35	28.2	NA	NA	NA	NA	NA	NA	NA	NA
Pietrantonio et al. (2016a, b)	40	50	21	52.5	71.4 (15/21)	23.8 (5/21)	0	4.8 (1/21)	0	0	0	0
Nummela et al. (2015)	19	48	12	63.2	58.3 (7/12)	41.7 (5/12)	0	0	0	0	0	0
Noguchi et al. (2015)	18	50	8	44.4	75.0 (6/8)	25.0 (2/8)	0	0	0	0	0	0
Sio et al. (2014)	10	236	4	40.0	50.0 (2/4)	50.0 (2/4)	0	0	0	0	0	0
Liu et al. (2014)	35	50	9	25.7	NA	NA	NA	NA	NA	NA	NA	NA
Alakus et al. (2014)	29	NA	20	69.0	11.1 (1/9)^a^	77.8 (7/9)	0	0	11.1 (1/9)	0	0	0
Singhi et al. (2014)	55	2	17	30.9	58.8 (10/17)	41.2 (7/17)	0	0	0	0	0	0
Nishikawa et al. (2013)	35	2	16	45.7	50.0 (9/18)^b^	44.4 (8/18)	5.6 (1/18)	0	0	0	0	0
Range	N	NA	NA	25.7–100.0	0–75.0	0–77.8	0–5.6	0–4.8	0–11.1	0–20.0	0–30.0	0–20.0
Median	NA	NA	NA	45.7	54.0	41.7	0	0	0	0	0	0

*NA* not available

^a^20 patients were reported to harbor *GNAS* mutation and variant forms of nine patients were described in detail by the author

^b^Eighteen variant forms were found in 16 patients

Taking the encoding of Gsα for example, once c.601C>T and c.602G>A mutation occur, the 201th amino acid residue, Arg, changes into cysteine (Cys) and histidine (His) respectively. The variants significantly alter the structure of GTPase domain in Gsα, and vastly decrease GTPase activity. As a consequence, Gsα fails to hydrolyze GTP and release phosphoric acid, remaining in activated status, which continuously stimulates downstream molecules (Fig. 3, blue dotted box).

## Influences of *GNAS* mutation to mucin secretion and cell proliferation

### Mucin expression in PMP

There are two major types of mucins, gel-forming mucins and transmembrane mucins (Johansson and Hansson 2016). Gel-forming mucins mainly include MUC2, MUC5AC, MUC5B, and MUC6. Transmembrane mucins mainly consist of MUC1, MUC3, MUC4, MUC12, MUC13, MUC16, and MUC17. A thorough review of the published literatures on mucin expression in PMP identified some distinctive features (Table 3). First, most researches focus on the expression status of gel-forming mucins, while little attention has been paid to transmembrane mucins. Second, MUC2 and MUC5AC are the most frequently expressed gel-forming mucins in PMP, with positive rates being 99.1% (314/317) and 96.5% (193/200), respectively, among the detected samples. MUC6 is rarely detected in PMP compared with MUC2 and MUC5AC, with positive rate of 12.5% (2/16). Third, the transmembrane MUC1 expresses variably in PMP, with positive rate being 41.3% (33/80). The expression status of MUC4 is currently unclear due to the limitation of sample number. Based on the available data from published literatures, it is advisable to focus more attention on in-depth study on MUC2 and MUC5AC.

**Table 3:** Mucin expression status in pseudomyxoma peritonei

References	Cases	Method	Gel-forming mucins (%)			Transmembrane mucins (%)	
			MUC2	MUC5AC	MUC6	MUC1	MUC4
Yan et al. (2019)	21	IHC	100	100	NA	NA	NA
Yan et al. (2020)	5	IHC	100	NA	NA	60	NA
Li et al. (2017a, b)	9	IHC	100	NA	NA	NA	NA
Li et al. (2017a, b)	8	IHC	100	NA	NA	NA	NA
Guo et al. (2011)	35	IHC	97.1	NA	NA	0	NA
Flatmark et al. (2010)	5	IHC	100	60.0	NA	0	100
Ferreira et al. (2008)	7	IHC	100	100	28.6	28.6	NA
Semino-Mora et al. (2008)	16	FISH	100	100	NA	NA	NA
McKenney and Longacre (2008)	1	IHC	100	NA	NA	NA	NA
Nonaka et al. (2006)	42	IHC	100	100	NA	NA	NA
Heiskala et al. (2006)	9	IHC	100	100	0	NA	NA
Bibi et al. (2006)	26	IHC	100	NA	NA	NA	NA
Mohamed et al. (2004)	33	IHC	100	NA	NA	84.8	NA
O’Connell et al. (2002a, b)	100	IHC	98.0	95	NA	NA	NA
Total	317	NA	99.1	96.5	12.5	41.3	100
Range	NA	NA	97.1–100	60.0–100	0–28.6	0–84.8	100–100
Median	NA	NA	100	100	14.3	28.6	100

*MUC2* mucin 2, *MUC5AC* mucin 5AC, *IHC* immunohistochemistry, *FISH* fluorescence in situ hybridization, *NA* not available

### *GNAS* functions on the regulation of mucin secretion

*GNAS* mutation is frequently detected in mucinous neoplasms of appendix (50%) and intraductal papillary mucinous neoplasm (IPMN) of pancreas (81%) (Furukawa et al. 2011; Wu et al. 2011), while the mutation rate in mucinous adenocarcinoma of colorectum, ovary, lung, and breast are relatively lower, even being 0% (Nishikawa et al. 2013). In addition, both PMP and IPMN share similar inertia biological behavior as well as hypersecretion of mucus. Therefore, it is inferred that *GNAS* might play some role in the regulation of mucin secretion (Alakus et al. 2014; Noguchi et al. 2015; Tokunaga et al. 2019).

The effect of *GNAS* mutation to mucin secretion has been proved by Nishikawa et al. (2013). The author transfected HT29 cells with an EF1a-GNAS^R201H^-IRES-Zeo plasmid. The result showed that cAMP, MUC2, and MUC5AC level elevated after the expression of GNAS^R201H^. While the application of PKA inhibitor downregulated the expression of *MUC2* and *MUC5AC* genes. Nishikawa’s study demonstrates that *GNAS* mutation might regulate mucin production through cAMP–PKA signaling pathway (Bradbury 2000; Jarry et al. 1994). The potential regulation method of cAMP–PKA signaling pathway might be stimulating cAMP-response element-binding protein (CREB) and activating transcription factor (ATF) family (Velcich and Augenlicht 1993). After entering nucleus, the activated CREB/ATF combines to the upstream cis-acting element of mucin genes and thus regulate mucin expression. Other studies have also proved that inhibitors of both PKA and heterotrimer G protein complex could also significantly downregulate mucin expression. Although *GNAS* mutation is proved to be an important promoter in mucin secretion of PMP, the current experiment was performed in colorectal cancer cell lines due to the difficulties in the culture of PMP tumor cells (Nishikawa et al. 2013). Besides, the influence of *GNAS* mutation to different types of mucin still needs further exploration.

The existed pathways which have cross reaction with cAMP–PKA pathway also participate in the regulation of mucin expression indirectly (Fig. 3): (1) MAPK signaling pathway. The activated cAMP influences MAPK signaling pathway via activating Ras or inhibiting Raf-1 by PKA. In pulmonary cystic fibrosis, it has been illustrated that hyperexpression of MUC2 was mainly regulated through Src/Ras/MAPK/pp90^rsk^ signaling pathway (Li et al. 1998). However, the function of Src/Ras/MAPK/pp90^rsk^ in PMP is not proven currently; (2) Ras–PI3K–Akt signaling pathway. PDE4B activated by this pathway functions as an antagonist against cAMP–PKA signaling pathway by clearing cAMP (Alakus et al. 2014); (3) PKC signaling pathway. Activated PKC has synergistic effect on cAMP–PKA pathway through activating Raf-1. Besides, Ca^2+^-dependent PKC-epsilon could also upregulate *MUC2* and *MUC5AC* expression (Hong et al. 1999).

### *GNAS* functions on the regulation of tumor cell proliferation

Generally, the current studies support the notion that PMP and colorectal cancer share similar gene mutation profiles, but vary vastly in mutation rate. PMP possesses higher mutation rates in *GNAS* and *KRAS*, while lower mutation rates in *TP53*, *APC*, and *PIK3CA* (Alakus et al. 2014; Tokunaga et al. 2019)*.* Nishikawa et al. transfected HT29 cells with an EF1a-GNAS^R201H^-IRES-Zeo plasmid. The cell proliferation remained the same, but accompanied with elevated mucin secretion. The result indicated that *GNAS* mutation mainly affect the expression level of mucin instead of tumor cell proliferation. *KRAS* is another important variant in PMP, and has been reported to promote tumor cell proliferation through the activation of MAPK signaling pathway (Alakus et al. 2014; Pylayeva-Gupta et al. 2011).

## Impacts of *GNAS* mutation to clinical–pathological characteristics and prognosis

### Correlation of *GNAS* mutation and clinical–pathological characteristics

In a study cohort of 55 patients, Singhi et al. (2014) demonstrated no significant association between *GNAS* mutation and gender, age, and adverse histological features (including cytologic grade, destructive invasion, tumor cellularity, angiolymphatic invasion, perineural invasion, and signet ring cells) (*P* > 0.05). However, the author found that *GNAS*-mutated PMP was prone to harbor concurrent *KRAS* mutation compared with GNAS-wild-type PMP (65% vs. 29%, *P* = 0.018).

Pietrantonio et al. (2016a, b) analyzed 15 patients with relapsed PMP, and revealed no association between *GNAS* mutation and gender, age, Eastern Cooperative Oncology Group performance status, histological grade, time elapsed from surgery to relapse, peritoneal cancer index (PCI), and completeness of cytoreduction. In another study of 40 PMP patients, Pietrantonio et al. (2016a, b) found that *GNAS* mutation was correlated to incomplete cytoreduction (*P* = 0.05) and *KRAS* mutation (*P* = 0.002). Besides, neither *GNAS* nor *KRAS* mutation were associated with pathological grade (*P* = 0.338 and 0.427, respectively).

From the studies by Pietrantonio et al. (2016a, b) and Singhi et al. (2014), it could be inferred that the presence of *GNAS* mutation is related to *KRAS* mutation. Considering the high incidence of these two variants in PMP and the statistically close relationship, the independent and synergistic effect as wells as the crosslink between *GNAS* and *KRAS* could be important issues to be explored in the mechanical studies of PMP.

Despite of the application of different criteria in histopathological classification, most of the studies showed that *GNAS* mutational status had no association with histopathological grade (Gleeson et al. 2018; Nummela et al. 2015; Pietrantonio et al. 2016a, b; Singhi et al. 2014). However, opposite opinions existed concerning the relation between *GNAS* mutation and histopathological grade. Noguchi et al. (2015) investigated mutation profiles of 18 PMP patients, revealing *GNAS* mutation in five low-grade PMP and three high-grade PMP. Noguchi hold the view that *GNAS* mutation might play a key role in both low-grade and high-grade PMP. On the contrast, in a study performed by Alakus et al. (Alakus et al. 2014), the result revealed that *GNAS* mutation rate is lower in high-grade PMP (21/23 vs. 1/6, *P* = 0.005). For the only patient with high-grade PMP presenting *GNAS* mutation, it was observed that the histopathology of the intraperitoneal implantation was a mixture of partly low-grade and partly high-grade PMP. Considering the existence of low-grade loci, Alakus et al. made a conclusion that high-grade PMP might not evolve from low-grade PMP.

### Impacts of GNAS mutation on PMP prognosis

Few studies were performed to investigate the association between *GNAS* mutation and prognosis of PMP. The results varied among different studies. Singhi et al. (2014) found that *GNAS* mutation did not affect the overall survival (OS) or time to disease progression. High tumor grade (AJCC G2 and G3) (*P* = 0.002) and lymph node involvement (*P* = 0.025) were associated with poorer OS. While HIPEC was associated with improved OS. Cox proportional hazard model identified that only lymph node involvement was the independent prognostic factor of PMP. In a study performed by Pietrantonio et al. (2016a, b), it was found that patients with *GNAS* mutation had significantly shorter median progression-free survival (PFS) than *GNAS*-wild type patients (5.3 months vs. not reached, *P* < 0.007). Later, in a study cohort of 40 patients, Pietrantonio et al. again demonstrated that *GNAS* mutation was associated with PFS. The other variables correlated to PFS were completeness of cytoreduction score, PCI score, and *KRAS* mutation status. However, multiple variate analysis revealed only PCI > 20 and *KRAS* mutation were the independent predictors of PFS.

## Summary

To sum up, *GNAS* mutation is one of the most important molecular biological features in PMP, which might function as promoting the secretion of mucin. The mutation sites of *GNAS* mutation is relatively stable, usually at Chr20: 57,484,420 (base pair: C-G) and Chr20: 57,484,421 (base pair: G-C). The presence of *GNAS* mutation results in the reduction of GTPase activity in Gsα, causing failure to hydrolyze GTP and release phosphoric acid, and eventually the continuous combining status of Gsα and GTP. The activated Gsα could thus continuously stimulate mucin secretion through the stimulation of cAMP–PKA signaling pathway. As presented above, there were already several studies proving that *GNAS* could elevate secretion level of mucin, but the experiments were limited in the cell lines of colorectal cancer. A more reliable evidence provided by experiments of genetic and protein level in PMP cell line is in urgent requirement.

The high mutation rate of *GNAS* in PMP patients has been observed about 10 years ago, when fresh tumor tissue or formalin-fixed, paraffin-embedded tissue was used for variant detection. However, the number of patients was limited, and most of the sequencings were non-whole-exome sequencing, which indicated the deficiency on the comprehensive view of PMP mutation profile. Generally speaking, the establishment of stable PMP cell line combined with comprehensive mutation profile would vastly help to improve the understanding of PMP genetically, and uncover the mechanism of PMP, especially the influence of *GNAS* mutation to mucin hypersecretion, which might eventually facilitate the innovation of new drugs targeting the molecules in the *GNAS*-related signaling pathways.

## Data Availability

All data and material generated or used during the study are available from the corresponding author by request.
